# A 3D Graphene Oxide Model Reveals Fine Particulate Matter Induced Cell Cycle Dysregulation in Neural Stem Cells

**DOI:** 10.3390/toxics14060536

**Published:** 2026-06-21

**Authors:** Siqi Li, Huiyun Chang, Mengjie Gao, Wenlou Zhang, Furong Deng, Fengge Chen, Xiaoman Zhu, Yu Song, Hong Zhang, Shaojie Liu, Ying Mu, Hui Ma, Ying Zhang

**Affiliations:** 1 College of Chemical and Pharmaceutical Engineering, Hebei University of Science and Technology, Shijiazhuang 050018, China; 2024105087@stu.hebust.edu.cn (S.L.); 2025105087@stu.hebust.edu.cn (M.G.); sjliu16@163.com (S.L.); 2 Hebei Key Laboratory of Intractable Pathogens, Shijiazhuang Center for Disease Control and Prevention, Shijiazhuang 050011, China; chy322516@163.com (H.C.); chenfengge319@163.com (F.C.); 13832120733@163.com (X.Z.); sy766479@163.com (Y.S.); zhsjzcdc@126.com (H.Z.); 3 Department of Occupational and Environmental Health Sciences, School of Public Health, Peking University, Beijing 100191, China; zhangwenlou@pku.edu.cn (W.Z.); lotus321321@126.com (F.D.); 4 State Key Laboratory of Industrial Control Technology, Research Centre for Analytical Instrumentation, Institute of Cyber-Systems and Control, Zhejiang University, Hangzhou 310027, China; muying@zju.edu.cn

**Keywords:** graphene oxide, scaffold, PM_2.5_, neural stem cell, proliferation, cell cycle

## Abstract

Fine particulate matter (PM_2.5_) exposure increases the risk of neurodevelopmental abnormalities by disrupting neural stem cell (NSC) proliferation and cell cycle homeostasis, which are critical for normal neurodevelopment. This study investigated the impact of fine particulate matter (PM_2.5_) on NSC proliferation and cell cycle using a three-dimensional (3D) graphene oxide (GO) scaffold that mimics the NSC microenvironment. PM_2.5_ exposure led to concentration-dependent decreases in NSC viability and induced G0/G1 phase arrest via the marked downregulation of Cyclin D1-Cdk4 and Cyclin E-Cdk2, which critically impact G1/S transition. NSCs in 3D GO scaffolds maintained higher expression of key cell cycle regulators (Cyclin A, Cdk1/Cdk2, APC, and Cdc20) and superior cell viability when suffering PM_2.5_ exposure, demonstrating the 3D culture environment was beneficial for NSC proliferation. We speculate that the 3D culture environment is more favorable and protective for cell proliferation. Therefore, these findings highlight the utility of the 3D GO scaffold for studying PM_2.5_ effects on growing neural stem cells. This work provides a physiologically relevant in vitro platform that captures microenvironment-dependent neurotoxic responses, consequently offering valuable mechanistic insights into PM_2.5_-induced developmental neurotoxicity.

## 1. Introduction

Despite overall improvements in recent years, air pollution, particularly fine particulate matter (PM_2.5_), remains a serious global threat to public health [1,2,3]. PM_2.5_ can be inhaled deep into the alveolar regions, traverse the alveolar capillary barrier to enter the systemic circulation, and thereby pose a potential threat to multiple organ systems, including the nervous system [4,5,6,7]. A growing body of toxicological evidence has consistently indicated that exposure to PM_2.5_ during early life, particularly during the fetal stage, is significantly associated with an increased risk of neurodevelopmental abnormalities in offspring, suggesting that the developing central nervous system exhibits heightened susceptibility to such environmental pollutants [7,8,9,10].

During the neurodevelopmental process, neural stem cells (NSCs), as the cornerstone of brain development, play a critical role in maintaining functional integrity [11]. NSCs are not only responsible for generating all major cell types of the central nervous system but also orchestrate the construction and repair of brain tissue through precisely regulated processes of proliferation, differentiation, and migration [12]. However, NSCs exhibit heightened sensitivity to external environmental stressors [13]. Once their homeostasis is disrupted, it may exert profound and lasting impacts on neurodevelopmental trajectories and may potentially even predispose individuals to neurological disorders later in life [14]. Therefore, investigating the disruptive effects of PM_2.5_ on NSC function is of critical importance.

Conventional two-dimensional (2D) cell culture models have been extensively utilized in toxicological research due to their operational simplicity and cost effectiveness. While these models have been instrumental in revealing the acute toxicity of various compounds, they possess a fundamental limitation in that they fail to recapitulate the complex three-dimensional (3D) microenvironment essential for the survival and function of NSCs within living brain tissue [15]. In vivo, NSCs reside within a specific “niche” [16] comprising an intricate extracellular matrix, diverse cell–cell contacts, and dynamic biochemical and physical signaling cues, all of which are significantly oversimplified on a 2D plane. In recent years, breakthrough progress in 3D organoid culture technology, such as the use of fully synthetic hydrogels (e.g., invasin-functionalized polyisocyanide hydrogel) as culture substrates, has enabled the creation of in vitro models that more closely mimic physiological conditions [17]. These systems excel in providing defined biochemical induction and mechanical support and have been successfully applied to the long-term culture of various epithelial organoids. However, for in vitro modeling of the nervous system, a core microenvironmental feature of electrical activity has not been adequately considered in these advanced synthetic matrices [18]. Electrical signaling between neural cells is fundamental in the formation and functionality of neural networks, and this critical characteristic should be taken into account when designing culture scaffolds.

Here, we developed and employed a 3D scaffold based on graphene oxide (GO) as an innovative platform for culturing NSCs. As a derivative of graphene, GO possesses not only excellent biocompatibility and ease of functionalization but also features a unique 3D interconnected porous architecture that closely mimics the physical topology of the extracellular matrix [19]. This structural characteristic effectively promotes the adhesion, migration, and 3D network formation of NSCs. Furthermore, the inherent electrical conductivity of GO enables it to provide neural cells with a biomimetic microenvironment capable of facilitating electrical signal transmission [20,21]. This capability represents a distinct advantage over most existing synthetic hydrogel-based systems.

Leveraging this advanced 3D GO culture model, our study systematically investigated the impact of PM_2.5_ exposure on NSC proliferative capacity and cell cycle progression. Our findings demonstrate that, compared to conventional 2D culture systems, the 3D GO scaffold not only provides a more physiologically relevant growth environment for NSCs but also significantly mitigates PM_2.5_-induced cytotoxicity. Further mechanistic investigation revealed that PM_2.5_ primarily triggers G0/G1 phase arrest in NSCs by downregulating the expression of core cell cycle regulatory proteins, specifically the Cyclin D1-Cdk4 and Cyclin E-Cdk2 complexes. Notably, this detrimental effect was substantially attenuated in the 3D GO environment. Collectively, our work not only deepens the understanding of the molecular mechanisms underlying PM_2.5_-mediated developmental neurotoxicity but also provides a novel technological platform and theoretical foundation for establishing more accurate and predictive in vitro models for neurotoxicological assessment.

## 2. Methods

### 2.1. PM_2.5_ Collection, Detection, and Extraction

We employed glass fiber filters to collect PM_2.5_ for polycyclic aromatic hydrocarbon (PAH) analysis, and quartz fiber filters to collect PM_2.5_ samples for metal and water-soluble inorganic ion analysis. Metals, including Sb, Al, As, Cd, Cr, Hg, Pb, Mn, Ni, and Se, were determined by ICP-MS (ELAN9000, PerkinElmer Inc., Waltham, MA, USA); water-soluble inorganic ions SO_4_^2−^, NO_3_^−^, Cl^−^, and NH_4_^+^ were analyzed by ion chromatography (ICS-900, Thermo Fisher Scientific Inc., Waltham, MA, USA); and 15 polycyclic aromatic hydrocarbons (acenaphthylene, acenaphthene, fluorene, phenanthrene, anthracene, fluoranthene, pyrene, chrysene, benzo[a]anthracene, benzo[b]fluoranthene, benzo[k]fluoranthene, benzo[a]pyrene, dibenzo[a,h]anthracene, benzo[g,h,i]perylene, indeno [1,2,3-cd]pyrene) were analyzed by GC-MS (Agilent 7890B-5977A, Agilent Technologies Inc., Santa Clara, CA, USA) after extraction with n-hexane and concentration.

The samples were extracted from the glass fiber filters by immersion in a mixture of methanol:deionized water (*v*/*v*, 4:1) and sonication for 60 min at room temperature (RT). The obtained extract was dried at 65 °C for 12 h. The dried samples were then resuspended in a serum-free culture medium containing 0.1% dimethyl sulfoxide (DMSO), diluted to 5 mg/mL, and stored at 4 °C before the experiment.

### 2.2. Characterization of GO Scaffold

GO scaffolds were prepared according to the method described in our previous work [22]. The scaffolds were sterilized at 121 °C for 15 min before the following experiments.

### 2.3. Swelling Test

The swelling ratio and swelling equilibrium of the GO scaffold were assessed by immersing the dry scaffold in PBS at 37 °C, consistent with standard cell culture conditions. Wet weights (W_wPBS_) were measured over 5 h after briefly placing the hydrated scaffold on filter paper to remove excess media. The formula for calculating the swelling percentage is shown in Equation (1).

Swelling % = [(W_wPBS_ − W_d_)/W_d_] × 100% (1)

Here, W_d_ is the initial dry weight. Data are presented as mean ± SD (*n* = 6).

### 2.4. Adsorption Experiment

The adsorptive property of the GO scaffold was evaluated using a model adsorption assay with a carbon black suspension. First, a standard curve was established for carbon black in PBS. A concentration gradient of standard working solutions (0, 0.05, 0.10, 0.15, 0.20, and 0.30 mg/mL) was prepared. All solutions were equilibrated at 37 °C for 2 h before analysis, with six replicates (*n* = 6) per concentration. Absorbance was measured using a UV-Vis spectrophotometer (UV1900, Yoke Instrument Inc., Shanghai, China). Following baseline correction, a full spectrum scan identified a characteristic absorption peak at 203 nm. Absorbance readings at 203 nm were then taken for each standard, and the average value per concentration was plotted against concentration (mg/mL) to generate a linear standard curve within the suitable range.

For the adsorption assay, pre-weighed dry GO scaffolds were allowed to reach swelling equilibrium in PBS. Each scaffold was incubated in 2 mL of 0.1 mg/mL standardized carbon black suspension at 37 °C. At predetermined time intervals (0, 15, 30, 60, 120, 240, 360, 720, 1440, 2880, and 4320 min), aliquots of supernatant were collected, centrifuged to remove particulates, and measured for absorbance at 203 nm. The decrease in absorbance, referenced against the calibration curve, was used to calculate the amount of carbon black adsorbed. Control experiments without scaffolds were conducted in parallel to account for non-specific particle settling.

### 2.5. Cell Culture

#### 2.5.1. Extraction of NSCs

In total, NSCs were derived from 30 11.5-day-old C57BL/6N mouse embryos of four pregnant mice; cultured on poly-L-lysine- (0.05 mg/mL, Solarbio, Beijing, China) and laminin (0.05 mg/mL, Sigma Chemical Co., St.Louis, MO, USA)-coated 4-well plates (Nalge Nunc International, Rochester, NY, USA) in a serum-free neurobasal medium (Gibco, Thermo Fisher Scientific Inc., Waltham, MA, USA); and supplemented with 1% glutamax (Gibco, USA), 1% penicillin/streptomycin (Solarbio, China), 2% B27 (Gibco, USA), and 10 ng/mL EGF (Gibco, USA) at 37 °C and 5% CO_2_. The primary NSCs were cultured for 7–10 days for proliferation and then, for differentiation, they were cultured in a neurobasal medium containing 1% fetal bovine serum (Gibco, USA) and supplemented with 1% glutamax, 1% penicillin/streptomycin, 2% B27, and 10 ng/mL EGF for 7 days for neuron and glial cell differentiation. All experiments in vitro had 3 replicates. All animal procedures were performed in accordance with the guidelines for animal ethics. This study was approved by the Shijiazhuang Center for Disease Control and Prevention’s Institutional Ethical Review Board Committee (#2020-5, 6 January 2020).

#### 2.5.2. 3D Culture of NSCs

Prior to cell culture, the GO scaffolds were soaked in neurobasal media for at least the swelling equilibrium time at 37 °C. NSCs were plated at a density of 1 × 10^5^ cells/well on GO scaffolds in a 4-well plate, cultured in serum-free neurobasal media, and supplemented with 1% glutamax, 1% penicillin/streptomycin, 2% B27, and 10 ng/mL EGF for at least 7 days for subsequent analysis.

### 2.6. Characterization of NSCs

#### 2.6.1. Identification of NSCs

Immunofluorescence analysis was performed for the identification of NSCs, neurons and glial cells. NSCs were cultured on sterilized glass coverslips coated with poly-L-lysine and laminin in 4-well plates for 3 days, fixed in 4% paraformaldehyde for 10 min at RT, and stained with nestin (10c2) Alexa Fluor 546 antibody (sc-23927, Santa Cruz Biotechnology, Santa Cruz, CA, USA) at 4 °C overnight. The neuronal and glial cells differentiated from NSCs were stained with rabbit anti-GFAP (#80788, Cell Signaling Technology, Danvers, MA, USA), anti-MAP2 (#8707, Cell Signaling Technology, USA), and anti-*β*-III Tubulin (#44456, Cell Signaling Technology, USA) at 4 °C overnight, and then stained with anti-rabbit IgG secondary antibodies conjugated to Alexa Fluor 488 and Alexa Fluor 555 for 1 h RT. Finally, the cells were stained with DAPI and the coverslips were mounted for visualization by fluorescence microscopy.

#### 2.6.2. Morphology of NSCs Cultured in GO Scaffolds

Scanning electron microscopy (SEM, SU-3500N, Hitachi Ltd., Tokyo, Japan) was used to analyze the surface morphology of GO scaffolds and NSCs cultured on GO scaffolds. The GO scaffold with NSCs was fixed in 4% paraformaldehyde for 1 h at RT and then dehydrated through graded concentrations of ethanol of 25%, 50%, 75%, 90%, and 100%. Subsequently, GO scaffolds with NSCs were heated at 65 °C for 1 h. Finally, GO scaffolds with NSCs were sputtered and coated with a thin layer platinum for analysis by SEM.

### 2.7. Cell Viability Assay

The Cell Counting Kit-8 (CCK-8) assay was used to analyze the viability of NSCs cultured in 2D and 3D environments. NSCs were cultured in 96-well plates with GO scaffolds (3D culture) and without GO scaffolds (2D culture as control group) at a density of 1 × 10^4^ cells/well. The NSCs were treated with PM_2.5_ concentrations (0, 10, 20, 50, 100, 200, and 300 μg/mL) for different times (24, 48, and 72 h). After exposure to different concentrations of PM_2.5_, the cells were treated with 10 μL CCK-8 reagent and incubated at 37 °C for 2–4 h. For viability of NSCs cultured in the 3D environment, before the cells were treated with 10 μL CCK-8 reagent, the GO scaffolds with NSCs were rinsed with the liquid in the 96-well plates, and then the GO scaffolds were removed from the wells. The absorbance of the 96-well plates was measured using a microplate reader at 450 nm (Biotek, USA).

### 2.8. Cell Cycle Assay

The cell cycle distribution was analyzed by flow cytometry using the DNA Content Quantitation Assay kit (CA1510, Solarbio, China). To investigate the effects of PM_2.5_ and 3D cell cultures of GO scaffolds on the cell cycle of NSCs, we established the 3D cell culture group without PM_2.5_ treatment (3D group), 3D cell culture with PM_2.5_ treatment group (3D + PM_2.5_ group), 2D cell culture group without PM_2.5_ treatment (2D group), and 2D cell culture with PM_2.5_ treatment group (2D + PM_2.5_ group). After exposure to PM_2.5_, NSCs were collected and resuspended at 1 × 10^6^ cells/mL and then fixed in 70% (*v*/*v*) ethanol at 4 °C overnight. The fixed cells were resuspended in RNase A solution and incubated at 37 °C for 30 min. Propidium iodide (PI) solution was then added and incubated at 4 °C in the dark for 30 min. The BD FACS Calibur instrument (BD Biosciences, USA) was used to measure the distribution of cells in different phases of the cell cycle, and the resulting data were analyzed using FlowJo software (version 7.6).

### 2.9. Western Blotting

After exposure to PM_2.5_, NSCs were collected and lysed in RIPA buffer. After homogenization and centrifugation, the supernatant was collected, and the protein concentration was determined using a BCA protein concentration kit (PC0020, Solarbio, China). Meanwhile, the supernatant was used to prepare the protein sample by boiling for 5 min. Proteins were separated by 12% SDS-PAGE and transferred to 0.22 μm PVDF membranes. Membranes were blocked with 5% BSA buffer and incubated with primary antibodies, Cyclin A (sc-53228, Santa Cruz, USA), Cyclin D1 (sc-8396, Santa Cruz, USA), Cyclin E (sc-377100, Santa Cruz, USA), Cdk2, Cdk4 (sc-23896, Santa Cruz, USA), Cdk1/Cdk2 (sc-53219, Santa Cruz, USA), APC (sc-53166, Santa Cruz, USA), Cdc20 (sc-53398, Santa Cruz, USA) and *β*-actin (sc-517582, Santa Cruz, USA) at 4 °C overnight, followed by incubation with HRP-conjugated secondary antibodies for 1 h at RT. The enhanced chemiluminescence assay kit was used to visualize the immunoproteins. Protein bands were normalized to *β*-actin and quantified using ImageJ software (version 1.48, National Institutes of Health, USA).

### 2.10. Statistical Analysis

Statistical analysis was performed using SPSS software (v21.0, IBM Corporation, Armonk, NY, USA). Data are presented as mean ± SD. For data that did not satisfy normal distribution, Spearman’s rank correlation analysis in nonparametric tests was used to determine differences between groups. For comparisons between multiple groups, one-way ANOVA was performed, followed by Fisher’s LSD post hoc test to identify specific group differences. A *p* < 0.05 was considered statistically significant.

## 3. Results

### 3.1. Detection of Components in PM_2.5_

The chemical characterization of PM_2.5_ collected from the sampling site is summarized in Table 1. The mean mass concentration of PM_2.5_ was 95 ± 43 μg/m^3^. Among the water-soluble inorganic ions, NO_3_^−^ and NH_4_^+^ were the most abundant, with concentrations of 16.95 ± 12.07 μg/m^3^ and 10.83 ± 6.88 μg/m^3^, respectively. Metal analysis revealed a distinct profile. Al was present at the highest concentration (267.14 ± 159.51 ng/m^3^), followed by Pb (59.09 ± 42.18 ng/m^3^) and Mn (43.46 ± 28.00 ng/m^3^). Trace elements of significant toxicological concern, including As (6.16 ± 5.56 ng/m^3^), Se (8.41 ± 7.71 ng/m^3^), and Cd (1.77 ± 1.88 ng/m^3^) were also detected. A total of 15 priority PAHs were quantified. The total PAH concentration was considerable, with high molecular weight (4–6 ring) PAHs dominating the profile. Benzo[k]fluoranthene (30.69 ± 17.36 ng/m^3^), benzo[b]fluoranthene (28.27 ± 14.33 ng/m^3^), and chrysene (23.33 ± 12.73 ng/m^3^) were the three most abundant congeners. The carcinogenic potency indicator, benzo[a]pyrene, was detected at a concentration of 8.90 ± 6.72 ng/m^3^.

### 3.2. Identification of NSCs in 2D Culture

**Figure 1:**
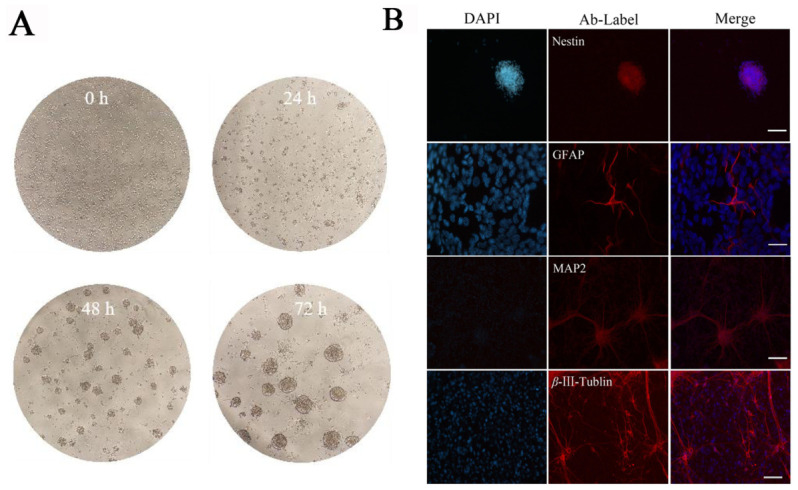
Identification of NSCs in 2D culture. (**A**) Morphological characterization of primary NSCs in culture (×200). (**B**) Immunofluorescence staining of DAPI (blue) and nestin (red, scale bar = 100 μm), GFAP (red, scale bar = 5 μm), MAP2 (red, scale bar = 5 μm), and *β*-III-Tubulin (red, scale bar = 20 μm) in NSCs. Figure had three replicates.

First, we examined the morphology of NSCs in a 2D culture system. The cells were derived from different embryos of the same pregnant mouse. As shown in Figure 1A, bright-field microscope images of NSCs illustrate their progression from single cells to neurospheres comprising dozens of aggregated cells. With increasing incubation time, the diameter of the neurospheres increased while their number gradually decreased. However, after 72 h of incubation, a reduction in central phototropism was observed in some neurospheres. Immunolabeling results in Figure 1B show the neurospheres comprised undifferentiated NSCs. Following the induced differentiation of NSCs, immunostaining with GFAP, MAP2, and *β*-III-Tubulin antibodies revealed that the cultured NSCs had the capacity to differentiate into both glial cells and neurons. These results demonstrated the successful isolation of NSCs not only maintained their undifferentiated state but also retained the potential for induced differentiation.

### 3.3. Swelling and Adsorption Properties of the GO Scaffolds

**Figure 2:**
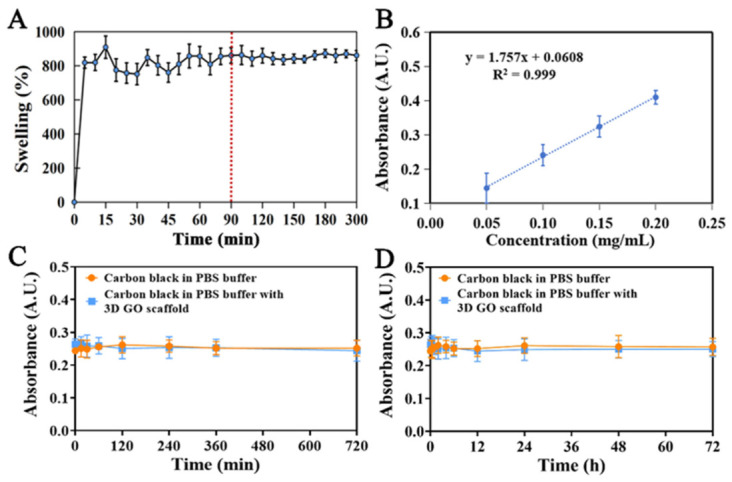
Swelling and adsorption properties of the GO scaffold. (**A**) Swelling kinetics of the scaffold in PBS, showing it reached equilibrium within approximately 90 min. (**B**) Standard absorbance curve for carbon black in PBS, with a linear range between 0.05 and 0.20 mg/mL. (**C**) Time-dependent absorbance of the carbon black solution incubated with or without the scaffold over 6 h. Data points represent the mean ± SD (*n* = 6). No significant adsorption was detected within this period. (**D**) Extended assay over 72 h confirmed the absence of measurable adsorption by the scaffold.

As shown in Figure 2A, the 3D GO scaffold exhibited excellent swelling properties. Its swelling ratio reached 800% within 15 min, indicating rapid and extensive swelling characteristics, and it essentially reached swelling equilibrium around 90 min. Therefore, in all subsequent experiments, the scaffold was allowed to swell fully before use to eliminate any interference from its own swelling process and ensure data reliability. To quantitatively evaluate the adsorption capacity of the 3D GO scaffold, this study employed carbon black as a model and established a standard absorbance curve for carbon black in PBS (Figure 2B). Since the curve was not linear below 0.05 mg/mL or above 0.20 mg/mL, a carbon black in PBS of 0.1 mg/mL was selected to investigate the scaffold’s adsorption performance. From the time-dependent absorbance curves in Figure 2C,D, it can be observed that the absorbance of the solutions at 0.1 mg/mL showed no significant difference between the groups with and without the 3D GO scaffold (Z = −0.23, *p* > 0.05). Quantitative analysis based on the standard curve in Figure 2B further confirmed that the 3D GO scaffold did not significantly alter the concentration of carbon black in the solution.

### 3.4. Culture of NSCs on 3D GO Scaffolds

**Figure 3:**
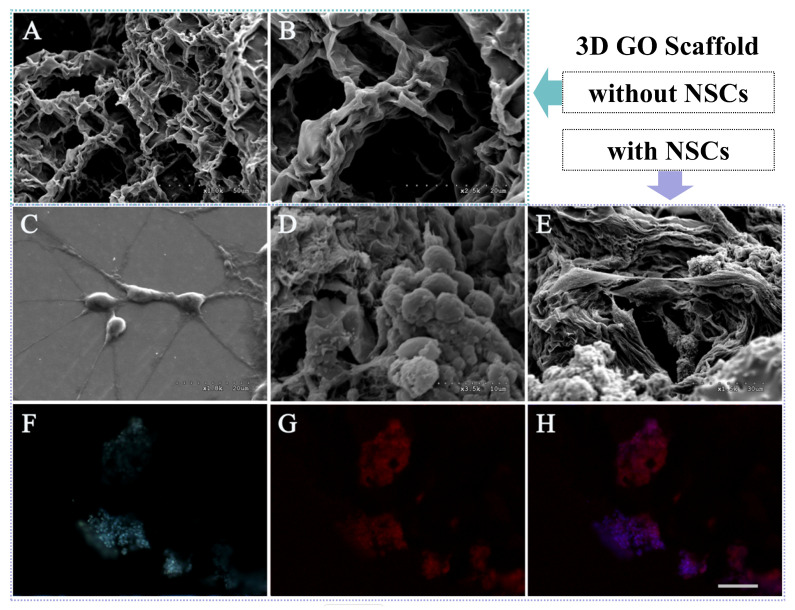
GO scaffolds used for NSC culture. (**A**,**B**) SEM analysis of the surface structure of the 3D GO scaffolds; (**C**) on the glass slide (scale bar = 20 μm); (**D**,**E**) on the 3D GO scaffolds (scale bar = 10 and 30 μm); and (**F**–**H**) immunofluorescence staining of DAPI (blue), nestin (red), and both (scale bar = 10 μm) of NSCs in 3D GO scaffolds. All experiments had three replicates.

In Figure 3A,B, the SEM images revealed that the fabricated GO scaffolds had an interconnected 3D porous architecture with uniformly distributed pores with diameters ranging from 30 to 50 μm. The NSCs were from different embryos of the same pregnant mouse. SEM analysis showed that the cells retained their characteristic neurosphere morphology while exhibiting a tendency to aggregate on the GO scaffolds. Notably, we observed NSCs growing along the scaffold matrix in Figure 3C,D, with cellular processes extending through the porous network and forming intricate intercellular junctions, resembling a “handshake” configuration between adjacent cells in Figure 3E. Figure 3F–H confirmed the presence of nestin-positive (red) NSCs throughout the scaffold architecture, with cells not only adhering to the surface but also penetrating deep into the porous matrix. This spatial distribution pattern, coupled with the observed cellular morphology and connectivity, strongly suggests that the 3D GO scaffolds provide an optimal biomimetic environment that supports NSC proliferation, migration, and network formation.

### 3.5. Impact of PM_2.5_ Exposure on NSC Proliferation

**Figure 4:**
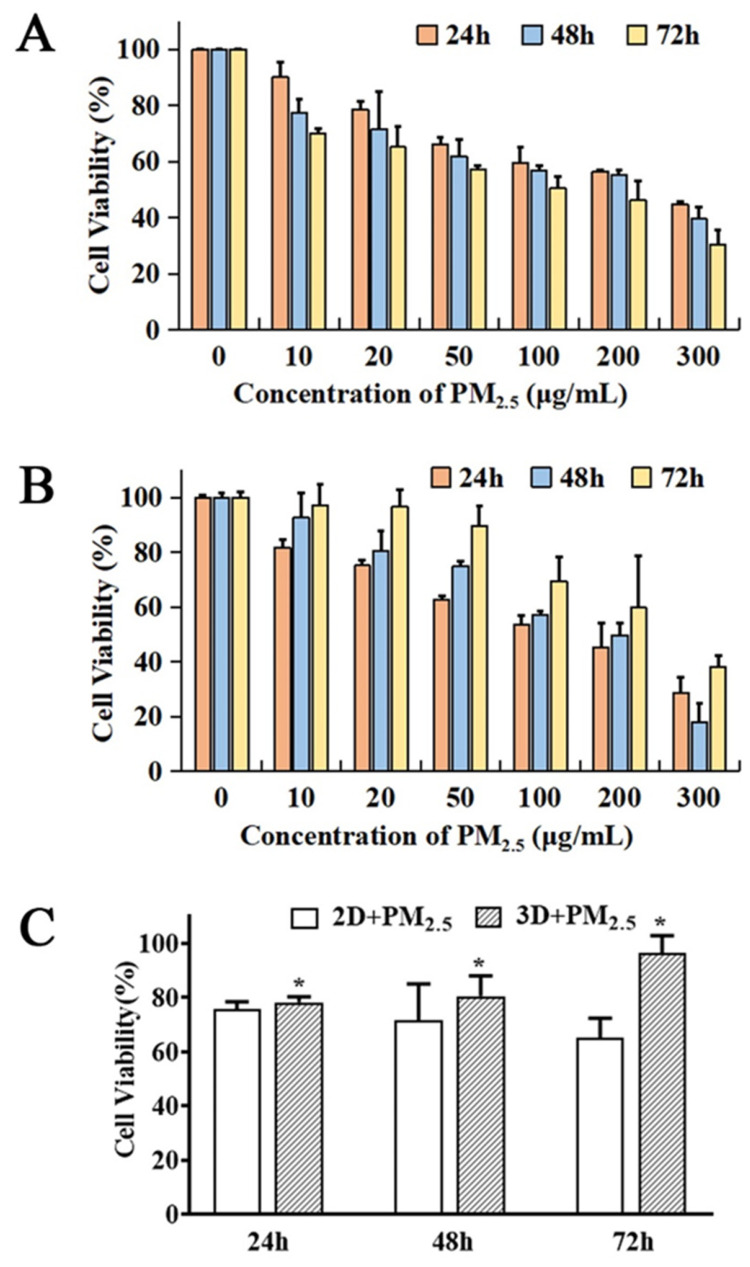
Effect of PM_2.5_ exposure on NSC viability. (**A**) Effect of different concentrations of PM_2.5_ exposure on cell viability of NSCs under 2D culture conditions. (**B**) Effect of PM_2.5_ exposure on cell viability of NSCs under 3D culture conditions. (**C**) Effect of 3D culture and 2D culture on cell viability of NSCs with 20 μg/mL (IC20) PM_2.5_ exposure. Data are presented as mean ± SD, *n* = 3 replicates. * *p* < 0.05 vs. control group.

As shown in Figure 4, cell viability results revealed a concentration-dependent decrease in NSC proliferation rate with increasing PM_2.5_ concentrations in both 2D and 3D cell cultures. In Figure 4A, all PM_2.5_-treated groups showed significant differences (* *p* < 0.05) compared to the control group (0 μg/mL). When cultured in 3D GO scaffolds, NSCs exhibited a similar concentration-dependent decrease in proliferation rate after PM_2.5_ exposure (*p* < 0.05). Interestingly, in contrast to the 2D culture system, NSC proliferation in the 3D environment displayed a time-dependent increase across all PM_2.5_ concentration groups. In Figure 4B, significant differences (* *p* < 0.05) were observed between 24 h and 72 h exposure, supporting that NSC viability in 3D culture exhibits a time-dependent recovery. Notably, at 20 μg/mL PM_2.5_ of inhibitory concentration 20% (IC20) value, NSCs maintained in 3D GO scaffolds showed significantly higher viability (*p* < 0.05) compared to 2D cultures (Figure 4C).

### 3.6. Effect of PM_2.5_ Exposure on NSC Cycle Progression

**Figure 5:**
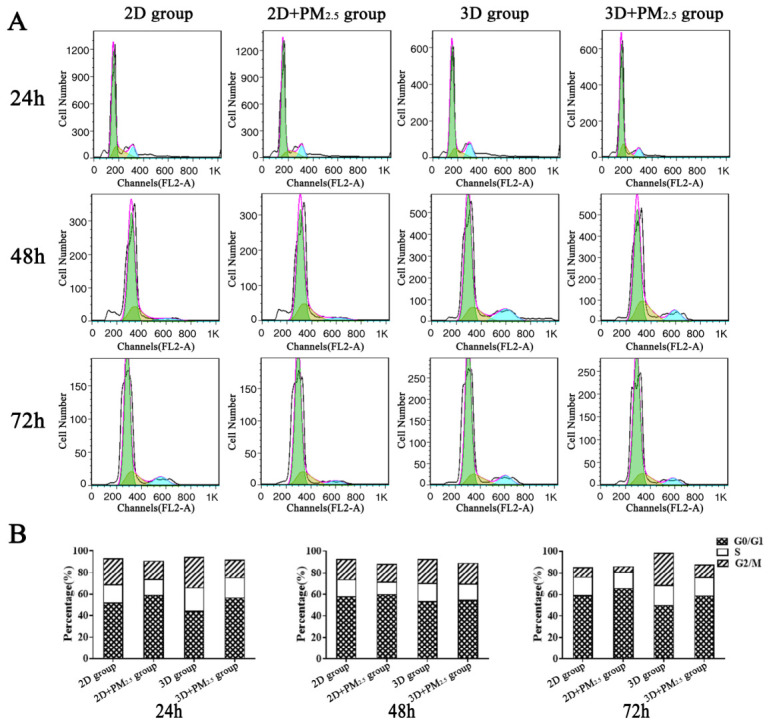
Cell cycle analysis of NSCs after PM_2.5_ exposure (24 h, 48 h, and 72 h) by flow cytometry. (**A**) Flow cytometry histograms showing cell distribution in G0/G1(green), S(yellow), and G2/M(blue) phases for 2D and 3D cultures, with and without PM_2.5_ exposure; (**B**) the percentage of cells in G0/G1, S, and G2/M phase for four groups: 2D, 2D + PM_2.5_, 3D, and 3D + PM_2.5_. Data are presented as means. All experiments had three replicates.

Subsequently, we cultured NSCs in both 2D and 3D GO scaffold culture systems, followed by PM_2.5_ exposure (20 μg/mL) for 24, 48, and 72 h. As shown in Figure 5, cell cycle analysis using flow cytometry revealed a time-dependent increase in the G0/G1 phase population, accompanied by a corresponding decrease in the S and G2/M phase populations in both culture systems. Notably, statistically significant increases in the G0/G1 phase were observed in a time-dependent manner (*p* < 0.05). The proportion of G0/G1 phase cells was significantly elevated at 48 and 72 h compared with 24 h in both culture systems.

**Figure 6:**
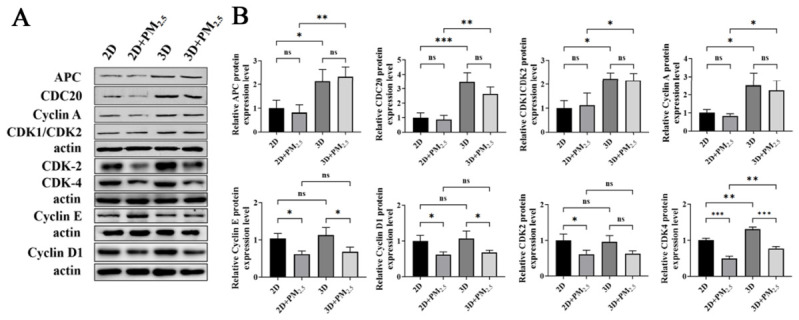
Expression of cell-cycle-related proteins by Western blot. (**A**) Expression of APC, Cdc20, Cdk1/Cdk2, Cyclin A, Cyclin E, Cyclin D1, Cdk4, and Cdk2 measured by Western blot. (**B**) Western blot band intensities were requantified. Data are presented as mean ± SD, *n* = 3; *p* < 0.05 is presented as *, *p* < 0.01 is presented as **, *p* < 0.001 is presented as ***, and non-significance (ns) is indicated by *p* > 0.05.

In Figure 6, Western blot analysis revealed that PM_2.5_ exposure significantly downregulated the expression of Cyclin E (*p* < 0.05), Cyclin D1 (*p* < 0.05), and Cdk4 (*p* < 0.001) in both 2D and 3D culture environments, while Cdk2 changed only in 2D culture (*p* < 0.05), suggesting PM_2.5_-induced proliferation inhibition. Interestingly, while Cdk4 expression was suppressed by PM_2.5_ exposure, the 3D culture environment appeared to mitigate this suppression. Furthermore, analysis of Cyclin A, Cdk1/Cdk2, APC, and Cdc20 expression patterns showed no significant changes after PM_2.5_ exposure. Nevertheless, their expression levels were significantly higher in the 3D culture environment than in 2D conditions.

## 4. Discussion

In this study, we applied GO scaffolds to 3D cultures of NSCs. Morphological and fluorescence labeling analyses of NSCs cultured in a 3D GO scaffold environment revealed their ability to form aggregated colonies both on the scaffold surface and within its porous matrix. Additionally, NSCs exhibited directional outgrowth along the scaffold architecture and established intercellular connections through extended cellular processes. Taken together, these observations suggest that the 3D GO scaffolds provide an in vivo like 3D microenvironment that supports NSC proliferation, differentiation, and network formation.

Crucially, we investigated how PM_2.5_ exposure impacted NNSC proliferation under 2D and 3D culture conditions. PM_2.5_ cytotoxicity analysis revealed a concentration-dependent decrease in NSC proliferation rate with increasing PM_2.5_ concentration. NSC proliferation in the 3D environment showed a time-dependent increase in all PM_2.5_ concentration groups at lower concentrations. The flow cytometry results showed that PM_2.5_ exposure prolonged the G0/G1 phase of NSCs, accompanied by a corresponding decrease in S phase and G2/M phase populations in both culture systems. To further investigate the molecular mechanisms underlying these observations, we examined cell cycle regulatory protein expression. In Western blot results, PM_2.5_ exposure significantly downregulated Cyclin D1 and Cdk4 expression, impacting G1 progression of NSCs. Cyclin D1 is a critical cell cycle regulator expressed during early G1 phase [23]. Its overexpression facilitates accelerated G1 to S phase transition by promoting rapid passage through the G1/S checkpoint and shortening the S phase duration [24,25]. Conversely, Cyclin D1 inhibition or knockdown induces cell cycle arrest [26,27]. The molecular mechanism involves Cyclin D1 binding to and activating Cdk4/6 to form Cyclin D-Cdk4/6 complexes [24]. These complexes play a pivotal role in promoting G1 phase progression and facilitating the G1/S transition [25]. Meanwhile, PM_2.5_ exposure also significantly downregulated Cyclin E (*p* < 0.05, in 2D and 3D cultures) and Cdk2 (*p* < 0.05, only in 2D culture), thereby impacting the subsequent process of cell cycle. Cyclin E, a pivotal regulator of late G1 phase progression, functions as the rate-limiting factor for the G1/S phase transition by forming an active complex with Cdk2 [28,29]. While Cyclin A expression remained stable, a decrease in Cdk2 levels (2D + PM_2.5_ vs. 2D, *p* < 0.05) potentially disrupted S phase progression through alterations in the Cyclin A-Cdk2 complex.

Disruption of Cyclin D1-Cdk4 signaling likely contributes to the subsequent dysregulation of Cyclin E-Cdk2 activity, ultimately leading to impaired cell cycle progression and inhibition of proliferation. This molecular alteration provides a mechanistic explanation for the observed cell cycle arrest in NSCs following exposure to PM_2.5_. Shen et al. demonstrated that PM_2.5_ exposure induced G0/G1 phase arrest in human umbilical vein endothelial cells, resulting in cell cycle dysregulation and arrhythmia [30]. Therefore, this abnormal proliferative activity may have profound implications for subsequent neural development and may contribute to long term neurological consequences.

There were no significant changes in the expression of APC, Cdc20, Cdk1/Cdk2 or Cyclin A between PM_2.5_-exposed groups and non-exposed controls. The expression levels of both APC and Cdc20 were unaffected by PM_2.5_ exposure. As APC-Cdc20 complexes are responsible for the ubiquitination and degradation of Cyclin B and Securin, the normal progression of this termination phase has been preserved; this stability ensures the completion of mitosis [31,32]. Additionally, a notable upregulation of these proteins expression was observed in 3D culture systems compared to 2D conditions. Cell viability results also revealed that the 3D culture environment was beneficial for NSC proliferation, even though there was PM_2.5_-induced cytotoxicity in NSCs. Therefore, we speculate that the 3D culture environment is more favorable and protective for cell proliferation.

## 5. Conclusions

In conclusion, this study establishes that acute PM_2.5_ exposure disrupts NSC proliferation by inducing G0/G1 phase cell cycle arrest, primarily through the downregulation of Cyclin D1-Cdk4 and Cyclin E-Cdk2 complexes, thereby impairing the G1/S transition. More significantly, we demonstrate that a 3D GO scaffold provides a biomimetic microenvironment for NSC growth; furthermore, we found that in the context of PM_2.5_-induced cytotoxicity, the 3D GO culture system enabled the maintenance of higher expression levels of critical cell cycle regulators (Cyclin A, Cdk1/Cdk2, APC, Cdc20). We speculate that the authentic 3D cell growth microenvironment may exert a protective effect compared to 2D culture. These findings provide crucial mechanistic insights into the developmental neurotoxicity of PM_2.5_, highlighting the limitations of 2D models and the paramount importance of the extracellular microenvironment in modulating cellular responses to environmental insults.

## Figures and Tables

**Table 1:** The main chemical compositions of PM_2.5_.

Item	Compound	Mean ± SD
PM_2.5_ (mg/m^3^)		0.095 ± 0.043
Water-soluble inorganic ions (μg/m^3^)	SO_4_^2−^	7.89 ± 4.58
	NO_3_^−^	16.95 ± 12.07
	Cl^−^	5.98 ± 4.26
	NH_4_^+^	10.83 ± 6.88
Metals (ng/m^3^)	Sb	3.47 ± 2.58
	Al	267.14 ± 159.51
	As	6.16 ± 5.56
	Cd	1.77 ± 1.88
	Cr	3.58 ± 2.00
	Hg	0.14 ± 0.11
	Pb	59.09 ± 42.18
	Mn	43.46 ± 28.00
	Ni	1.72 ± 1.08
	Se	8.41 ± 7.71
PAHs (ng/m^3^)	Acenaphthylene	0.34 ± 0.66
	Acenaphthene	0.09 ± 0.00
	Fluorene	2.74 ± 1.95
	Phenanthrene	7.37 ± 5.36
	Anthracene	10.30 ± 6.71
	Fluoranthene	12.05 ± 5.26
	Pyrene	17.86 ± 9.29
	Chrysene	23.33 ± 12.73
	Benzo[a]anthracene	20.15 ± 10.85
	Benzo[b]fluoranthene	28.27 ± 14.33
	Benzo[k]fluoranthene	30.69 ± 17.36
	Benzo[a]pyrene	8.90 ± 6.72
	Dibenzo[a,h]anthracene	5.76 ± 8.12
	Benzo[g,h,i]perylene	7.69 ± 5.38
	Indeno[1,2,3-cd]pyrene	5.90 ± 5.92

## Data Availability

The original contributions presented in this study are included in the article. Further inquiries can be directed to the corresponding authors.
